# Cold Atmospheric Plasma Induces Growth Arrest and Apoptosis in Neurofibromatosis Type 1-Associated Peripheral Nerve Sheath Tumor Cells

**DOI:** 10.3390/biomedicines12091986

**Published:** 2024-09-02

**Authors:** Brian Na, Blake Haist, Shilp R. Shah, Graeme Sabiston, Steven J. Jonas, Jeremie Vitte, Richard E. Wirz, Marco Giovannini

**Affiliations:** 1 Department of Head and Neck Surgery, UCLA David Geffen School of Medicine, Los Angeles, CA 90095, USA; bna@mednet.ucla.edu (B.N.); shilpshah14@ucla.edu (S.R.S.); jvitte@mednet.ucla.edu (J.V.); 2 UCLA Neuro-Oncology Program, Department of Neurology, UCLA David Geffen School of Medicine, Los Angeles, CA 90095, USA; 3 Jonsson Comprehensive Cancer Center, UCLA David Geffen School of Medicine, Los Angeles, CA 90095, USA; sjjonas@mednet.ucla.edu; 4 Samueli School of Engineering, UCLA, Los Angeles, CA 90095, USA; haistb@oregonstate.edu (B.H.); graemes1@ucla.edu (G.S.); richard.wirz@oregonstate.edu (R.E.W.); 5 College of Engineering, Oregon State University, Corvallis, OR 97331, USA; 6 California NanoSystems Institute, UCLA, Los Angeles, CA 90095, USA; 7 Division of Pediatric Hematology Oncology, Department of Pediatrics, UCLA David Geffen School of Medicine, Los Angeles, CA 90095, USA

**Keywords:** neurofibromatosis type 1, cancer therapy, cold atmospheric plasma, reactive oxygen species, reactive nitrogen species, apoptosis

## Abstract

Neurofibromatosis type 1 (NF1) is an autosomal dominant disorder resulting from mutations in the *NF1* gene. Patients harboring these mutations are predisposed to a spectrum of peripheral nerve sheath tumors (PNSTs) originating from Schwann cells, of which malignant peripheral nerve sheath tumors (MPNSTs) are the deadliest, with limited treatment options. Therefore, an unmet need still exists for more effective therapies directed at these aggressive malignancies. Cold atmospheric plasma (CAP) is a reactive oxygen species (ROS) and reactive nitrogen species (RNS) generating ionized gas that has been proposed to be a potential therapeutic modality for cancer. In this study, we sought to determine the effects of CAP on NF1-associated PNSTs. Utilizing established mouse and human cell lines to interrogate the effects of CAP in both in vitro and in vivo settings, we found that NF1-associated PNSTs were highly sensitive to CAP exposure, resulting in cell death. To our knowledge, this is the first application of CAP to NF1-associated PNSTs and provides a unique opportunity to study the complex biology of NF1-associated tumors.

## 1. Introduction

Neurofibromatosis type 1 (NF1) is an autosomal dominant disorder stemming from mutations in the *NF1* gene on chromosome 17q11.2, which leads to a hyperactive RAS signaling pathway. Affecting 1 in 3000 individuals worldwide, approximately 50% of NF1 patients develop plexiform neurofibromas (PNs), a histologically benign heterogeneous nervous sheath tumor arising from Schwann cells that display harbor a biallelic *NF1* inactivation. PNs can be painful and disfiguring, adversely impacting quality of life. Of greater concern are the 8–15% of PNs that can spontaneously transform into malignant peripheral nerve sheath tumors (MPNSTs), which are aggressive cancers with a dismal prognosis [1]. Despite maximal multimodal therapies, including chemotherapy, surgery, and radiotherapy, MPNSTs are the primary driver of mortality in NF1 patients who lack reliable therapeutic options [2]. Surgery is the primary treatment for these tumors, but it can be challenging depending on their anatomical location, and complete resection is generally recommended to prevent tumor regrowth [3]. Despite the recent approval of selumetinib, a MEK inhibitor for symptomatic and inoperable PNs, it is not curative and it is unknown whether MEK inhibition prevents transformation of PNs to MPNSTs [4].

**Figure 1:**
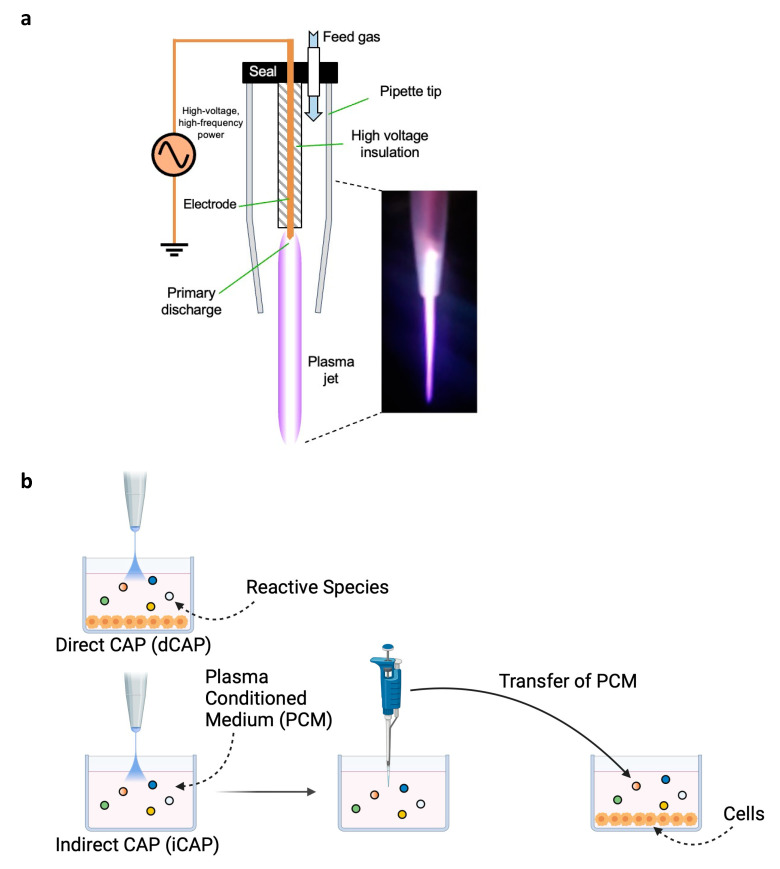
Cold atmospheric plasma (CAP) delivery schematic. (**a**) Diagram of CAP delivery device attached to a power supply with helium gas fed in a seal. Electrodes create an electrical charge difference that create CAP containing reactive oxygen and nitrogen species that exert their effects on the target of choice. (**b**) Two main accepted methods of delivery: Direct CAP, where cells and medium are concurrently exposed to CAP; and Indirect CAP, where the culture medium only is exposed to CAP to create a Plasma Conditioned Medium that is then transferred to a cell-containing well.

Cold atmospheric plasma (CAP) offers a new solution for selective anticancer therapy, where a noble gas such as helium is fed into a high-voltage environment, leading to an ionized gas (Figure 1) [5]. Existing in a state of non-thermal equilibrium at room temperature, CAP contains reactive oxygen and nitrogen species (RONS) that can be applied to living tissues. CAP has shown promise in many aggressive and difficult-to-treat cancers either directly to the tissue or indirectly via an intermediate medium [6,7]. In the context of solid tumors, CAP has exhibited efficacy across diverse types, even in recurrent cases where traditional multimodal therapies have limited effectiveness [8,9]. Furthermore, it has been postulated that CAP could offer more selective cytotoxicity in cancer cells compared to normal cells [10].

Despite its promise as a potential therapeutic option, to date, CAP has not been tested in NF1-associated peripheral nerve sheath tumors (PNSTs), including PNs and MPNSTs. Due to the prevalence of these lesions in patients with NF1 and that not all PNs can be surgically excised, it is crucial to explore and develop alternative novel treatment modalities. Therefore, we hypothesized that CAP could also be applied to NF1-associated tumors.

To investigate the role of CAP in NF1-associated PNSTs, we conducted a dose–response assessment of CAP exposure in multiple established NF1-associated cell lines. We report that NF1-associated cell lines have a dose–response effect with CAP exposure, providing novel clues for its mechanism of action. We also demonstrate the in vivo efficacy of CAP exposure for MPNST xenografted tumors, demonstrating that CAP exposure could be a promising therapeutic solution for NF1 patients.

## 2. Materials and Methods

**CAP device and treatment.** A single electrode found within a plastic tapered shell formed from a pipette tip was used as a delivery tool [11,12,13]. The shell directs the flow of high-purity helium down the length of the electrode to the tip, where it was ionized, and out to the target (Figure 1). The electrode, formed from sharpened copper wire, was supplied with a 1.4 kV peak-to-peak sinusoidal voltage oscillating at 52 kHz. The electrode had a thin insulating coating above the tip. Helium flow was set to 1.21 L/min throughout the experiments. The electronic signal supplied to the electrode was generated using custom step-up electronics in combination with a BK Precision 4013 DDS Sweep Function Generator (BK Precision Corporation, Yorba Linda, CA, USA) and a CPX400DP—Dual 420 Watt PowerFlex DC PSU (Aim-TTi, Cambridgeshire, England, UK). The tip of the CAP shell was placed about 1 cm above the sample throughout the experiments. Two experimental delivery methods were tested: (1) direct CAP (dCAP), where both cells and medium are exposed to CAP together, and (2) indirect CAP (iCAP), where the culture medium only is exposed to CAP to create a plasma-conditioned medium (PCM), which is then transferred to cell-containing wells (Figure 1). 

**Cell lines and culture.** The ESC-FC1208NF1 (*Nf1*^−/−^) and ESC-FH1208NF1 (*Nf1*-WT) cell lines were derived from *Nf1^flox^*^/*flox*^ mouse embryonic primary Schwann cell cultures that were (ESC-FC1208NF1) or were not (ESC-FH1208NF1) infected with Adenovirus-Cre and cultured in N2 medium, as previously described [14,15]. The NF1-mutated plexiform neurofibromas (CRL-3387, CRL-3389, CRL-3390) cell lines, human NF1^+/−^ (CRL-3391), and NF1 WT (CRL-3392) Schwann cell lines were purchased from ATCC through the Neurofibromatosis Therapeutic Acceleration Program (NTAP) [16]. The 88-14 and STS26T MPNST cell lines were generously gifted by Dr. Nancy Ratner at Cincinnati Children’s Hospital Medical Center; the S462TY cell line was generously provided by Dr. Timothy Cripe at Nationwide Children’s Hospital [17]. Cells were cultured in Dulbecco’s modified Eagle’s medium (DMEM) (Invitrogen, Waltham, MA, USA) with 10% fetal bovine serum (FBS) (Invitrogen) and 1% penicillin/streptomycin (100 U/mL; Invitrogen) at 37°C in 5% CO_2_. 

**in vitro cell viability.** Cell lines were seeded in a 96-well plate at a density that would lead to 70–80% confluence per well at 48 h after CAP exposure. Cells were then treated with dCAP and iCAP for 10, 30, 60, 90, and 180 s and were further incubated for another 48 h. Cell viability was measured using the ATPlite 1-Step Luminescence Assay System (Perkin-Elmer, Boston, MA, USA) following the manufacturer’s protocol. Each experimental condition was tested in triplicate, and results were normalized to untreated wells. 

**Assessment of apoptosis and necrosis.** Cells were seeded in 200 µL of growth medium in white 96-well microtiter plates at the concentration of 6000 cells per well in 4 technical replicates. Control wells with cell culture medium only to determine background luminescence and background fluorescence were included. In addition, control wells with cells without CAP exposure were also included. In a separate 96 well plate, 200 µL of CAP was exposed for 180 s, and then 100 µL were transferred to the cell-containing wells. Serial dilution, per manufacturer’s instructions, was then performed. In total, 50 µL of 2× concentrated RealTime-Glo™ Annexin V Apoptosis and Necrosis Detection Reagent (ProMega, Madison, WI, USA) in growth medium was added to each well. Cells were incubated in a covered 96-well assay plate at 37 °C 5% CO_2_ in a humidified cell culture incubator. Luminescence and fluorescence (Ex 485, Em 525) was performed on the GloMax^®^ Discover instrument (Promega, Madison, WI, USA) using the RealTimeGlo™ Annexin V Apoptosis Assay every hour for 72 h. 

**Flow cytometry analysis for cell cycle.** Cells were fixed in 70% ethanol in phosphate-buffered saline (PBS) for 30 min. After staining with 40 μg/mL of propidium iodide (PI, Sigma-Aldrich Chemical Co., St. Louis, MO, USA) for 30 min, the phase distribution of the cell cycle was determined by using a flow cytometer and was analyzed with Attune NxT (ThermoFisher Scientific, Waltham, MA, USA) and ImageStreamISX (Millipore Sigma, Burlington, MA, USA).

**MPNST xenograft mouse model and CAP treatment.** Female *nu*/*nu* mice (4 to 5 weeks old) were purchased from the Charles River Laboratories. The subcutaneous *NF1^−^^/−^* S462TY human MPNST xenograft model has been previously described [18]. The iCAP method was evaluated on mice using the S462TY MPNST cell line xenografted subcutaneously into their flank (1 × 10^7^ cells). Mice weight and tumor volume measurements were taken 3 times per week. Volume was assessed using perpendicular measurements with a caliper and was calculated using the formula: Length × Width^2^ × (π/6). Tumors were allowed to establish until their volume reached 100 mm^3^, at which point CAP exposure was performed. The mice were randomized to the control or iCAP groups based on tumor volume by ordering them from largest to smallest volume to ensure there was no statistically significant difference between the two groups (Figure S1). The mice were anesthetically induced by inhalation of isoflurane. For iCAP exposure, 200 µL of PBS was exposed for 180 s in a 96-well plate, and 100 µL of the CAP-exposed medium was immediately injected in the middle of the tumor mass. For control mice, 100 µL of PBS after 180 s of helium exposure was injected in the middle of the tumor mass. Weight and tumor volume measurements were taken from mice during approximately four weeks post-exposure before euthanasia and excision of the tumor. Tissues were fixed for 24 h in formalin and embedded in paraffin, following standard protocols.

**Cleaved caspase 3 histologic staining.** Paraffin-embedded sections were cut at 4 μm thickness. The paraffin was subsequently removed with xylene and rehydrated through graded ethanol. Endogenous peroxidase activity was blocked with 3% hydrogen peroxide in methanol for 10 min. Heat-induced epitope retrieval (HIER) was carried out for all sections in AR9 buffer (AR9001KT, Akoya Biosciences, Menlo Park, CA, USA) using a Biocare Decloaker at 95 ^o^C for 25 min. The slides were then stained with Cleaved Caspase-3 (Asp175) (#9661, Cell Signaling, Boston, MA, USA) at room temperature for 1 h. The Cleaved Caspase-3 signal was detected using the Dakocytomation Envision + System Labelled Polymer HRP anti-rabbit (K400311-2, Agilent, Santa Clara, CA, USA). All sections were visualized via the diaminobenzidine reaction and counterstained with hematoxylin.

**Ki67 histologic staining.** For Ki67 staining, automated detection was performed using a Leica Bond RX processor with the following steps based on Protocol F using the Bond Polymer Refine Detection kit (DS9800, Leica Biosystems, Deer Park, IL, USA). Briefly, the “Bake and Dewax” protocol on the Leica BOND System was utilized. Subsequently, HIER using the ER2 (BOND Epitope Retrieval Solution 2, Leica Biosystems, Cat# AR9640) buffer was performed at 100 °C for 20 min. After a 5 min peroxide block, slides were washed with BOND wash buffer three times followed by a 60 min incubation with the Ki67 primary antibody (Ab 92742, Abcam, Cambridge, UK). BOND wash buffer was applied three times in conjunction with a 10 min incubation period with Dakocytomation Envision System Labelled Polymer Horseradish Peroxidase (HRP) anti-rabbit (K400311-2, Agilent, Santa Clara, CA, USA). After incubation with the polymer, BOND wash buffer was placed on the slides five times, washed with deionized water, and incubated with Mixed DAB Refine for 10 min. Slides were subsequently washed with deionized water three times, incubated with hematoxylin for 10 min, washed with BOND wash buffer three times, and then washed again with deionized water. Slides were then dehydrated in series of alcohols, cleared with histoclear, and mounted with Permount.

**Histological analysis.** Cleaved Caspase 3 and Ki67 slides were scanned and analyzed using digital pathology software QuPath 0.4.4. The area of quantification for each slide was manually selected to cover the whole tissue area while avoiding analysis of necrosed regions and artifacts. The positive cell detection function was utilized for determining the percentage of stained cells. Parameters were set up for the optical density sum with a pixel size of 0.5 µm and a background radius of 8 µm. The single intensity threshold was set at 0.4 for Cleaved Caspase 3 and 0.2 for Ki67, the sigma level was 1.5 µm, and the cell area range was from 10 µm^2^ to 400 µm^2^.

**Statistical Analysis.** All results are presented as means ± SEM. Tukey post hoc tests and one-way or two-way analysis of variance (ANOVA) tests were used for multiple comparisons (when more than two groups or 2 variables were compared). Student’s t-test was used for comparisons between two groups. All statistical analyses were carried out with the Prism software package (Prism 10.2.3, GraphPad Software, 2024). The threshold for statistical significance was *p* ≤ 0.05.

## 3. Results

**NF1-associated tumor cell lines are sensitive to CAP**. We sought to explore the efficacy of CAP in NF1-associated tumors. As such, to study the impact of CAP on NF1-associated tumor cell death, we first evaluated cell death in vitro at different CAP exposure times at 10, 30, 60, 90, and 180 s, utilizing dCAP and iCAP (Figure 1b) [19].

**Figure 2:**
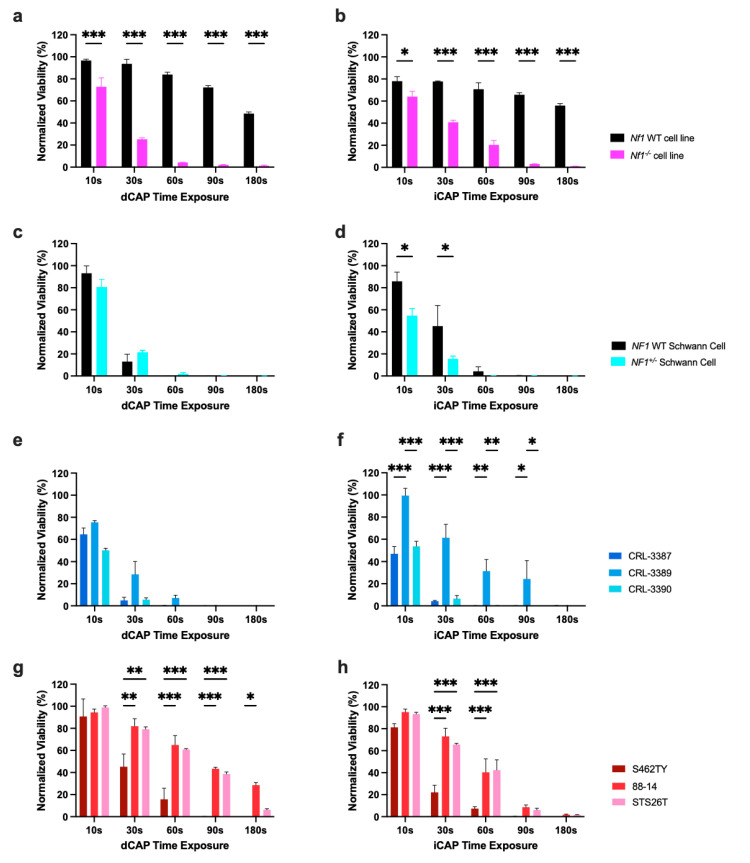
Increased CAP exposure time decreases cell viability. Cells were plated in triplicate, and 2 days later were exposed to plasma for 10 to 180 s. ATPlite 1-step assay was performed 48 h after exposure to assess cell viability normalized to untreated wells. NF1 mouse Schwann cell lines (**a**,**b**), human *NF1^+/−^* (CRL-3391) and NF1 WT Schwann cell lines (CRL-3392) (**c**,**d**), patient derived NF1-mutated plexiform neurofibromas (CRL-3387, CRL-3389, and CRL-3390) (**e**,**f**), and 88-14 and S462TY patient-derived NF1 MPNST cell lines, STS26T sporadic MPNST (**g**,**h**) were exposed to direct CAP treatment (dCAP) (**a**,**c**,**e**,**g**) or indirect CAP treatment (iCAP) (**b**,**d**,**f,h**). (*: *p* ≤ 0.05, **: *p* ≤ 0.001, ***: *p* ≤ 0.001).

Cell viability was assessed at 48 h after CAP exposure using the ATPlite 1-Step Luminescence Assay and was normalized to untreated controls. Across all NF1 cell lines, there was a progressive dose–response effect with lower cell viability at longer CAP exposure, and there was no difference between dCAP and iCAP (Figure 2). Furthermore, we observed a significant lower viability of the mouse *NF1^−^*^/*−*^ compared to the mouse *NF1*-wild-type (WT) Schwann cell lines (Figure 2a,b). 

We next assessed the role of CAP on human Schwann cells, the cell of origin for NF1-associated PNST in patients. We observed that the *NF1*-heterozygous human Schwann cells is more sensitive to iCAP treatment than *NF1*-WT human Schwann cells (Figure 2c,d). Cell viability of three NF1-associated PNs with different *NF1* genetic mutations was also affected by CAP treatment, although it showed individual differences in the extent of sensitivity (Figure 2e,f). Finally, three MPNST cell lines showed sensitivity to both dCAP and iCAP, with difference in response observed between the two NF1-associated MPNSTs (S462TY and 88-14), likely due to tumor heterogeneity (Figure 2g,h). In sum, CAP induces a reduction in cell viability, in a dose-dependent manner, in NF1-associated PNSTs. 

**Figure 3:**
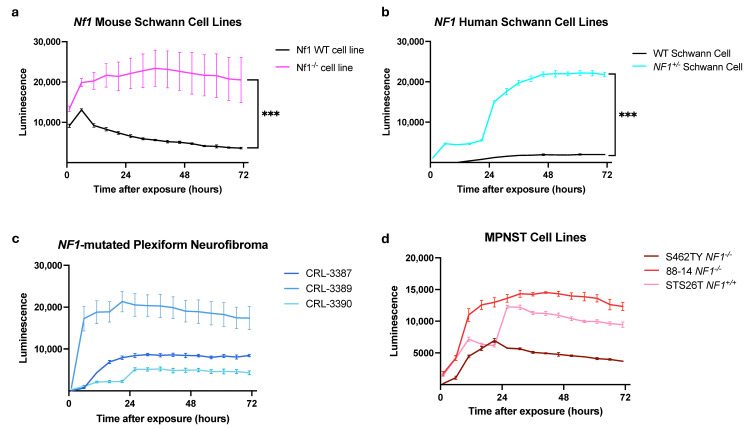
iCAP induces apoptosis in NF1-mutated cell lines and was obtained in quadruplicate. (**a**) The ESC-FC1208NF1 (*NF1^−^*^/*−*^) and ESC-FH1208NF1 (NF1 wild-type) mouse Schwann cell lines. (**b**) *NF1 ^+^*^/*−*^ Schwann cell and NF1 wild-type Schwann cell. (**c**) Patient-derived NF1-mutated plexiform neurofibromas (**d**). NF1-associated MPNSTs (S462TY and 88-14) and a sporadic MPNST (STS26T). (***: *p* ≤ 0.001).

**iCAP induces apoptosis in NF1-associated PNSTs.** Many studies have demonstrated that CAP exposure induces apoptosis of different tumor types [6]. However, to date, no study has investigated the mechanism of CAP-induced cell death in NF1-associated PNSTs. To gain insight into how CAP affects PNST cells, NF1-associated PNST cell lines were exposed to 180 s of iCAP, and quantitative analysis was conducted utilizing the RealTime-Glo Annexin V Apoptosis and Necrosis Assay. We observed that across all cell lines, tumor cell death after CAP treatment occurred mainly through apoptosis rather than necrosis, as validated with a positive control (Staurosporine) and a negative control (no CAP exposure) (Figure 3, Figures S1 and S2). Interestingly, we found that the mouse *Nf1^−^*^/*−*^ and the human *NF1^+^*^/−^ Schwann cell lines were more sensitive to CAP-induced apoptosis than the corresponding wild-type cell lines (Figure 3c,d)**.** In sum, our data suggest that CAP mediates cell death through apoptosis in NF1-associated PNSTs.

**Figure 4:**
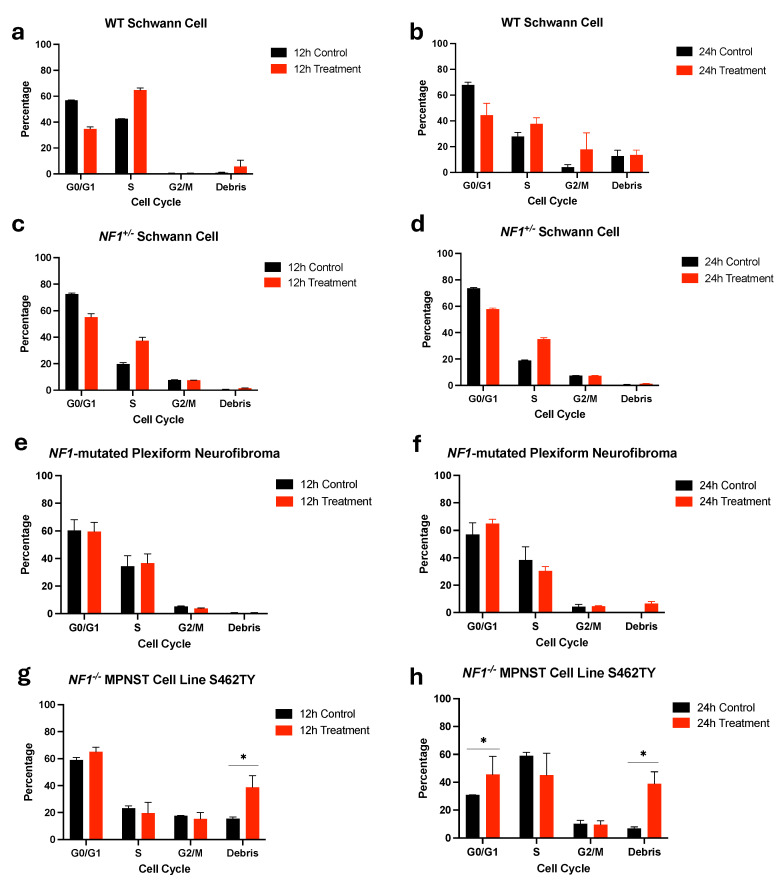
iCAP induces cell cycle arrest and death in NF1-associated MPNSTs. Triplicates were performed. Cell culture media was exposed to CAP for 60 s, and PCM was then transferred to cell-containing wells. Cell cycle analysis was performed at the 12 h (**a**,**c**,**e**,**g**) and 24 h mark (**b**,**d**,**f**,**h**). Cells were arrested in the G1 phase for the S462TY cell line at the 24 h mark (*: *p* < 0.05).

**iCAP treatment induces cell cycle arrest.** We conducted a cell cycle analysis at the 12 and 24 h timepoints to explore the mechanism of CAP-induced anti-proliferative effects across NF1-associated PNSTs after 60 s of CAP exposure (Figure 4 and Figure S3–S7). For the *NF1^+^*^/*−*^ Schwann cell, wild-type Schwann cell, and NF1-associated plexiform neurofibroma (CRL-3387), there was no significant difference in all cell cycle phases after CAP exposure (Figure 4a–f). For the NF1-associated MPNST cell line S462TY, there were proportionally more cells in the G1 phase at the 24 h mark, suggesting that cells arrested in the G1 phase are prone to apoptosis, cell death, and increased cellular debris (Figure 4h). To ensure that these findings were not due to artifacts, cell imaging was performed in parallel with no aggregates or mislabeled cells observed (Figure S3). 

**Figure 5:**
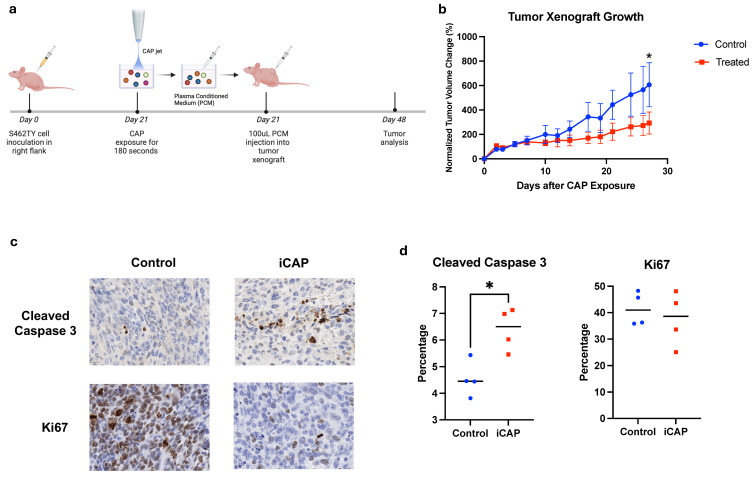
iCAP inhibits MPNST tumor growth in a mouse xenograft model. (**a**) S462TY cells were subcutaneously injected into the right flank of nu/nu mice. In total, 100 μL of PCM (*n* = 4) or PBS (*n* = 4) were then injected into the tumor once xenografts were established (Day 21). Tumors were monitored and analyzed at Day 48. (**b**) Tumor volume change over monitoring time, normalized to time of treatment. (**c**) Representative IHC images of xenograft tumors for cleaved Caspase 3 and Ki67. (**d**) Percentage staining for Caspase 3 and Ki67 in CAP and control was assessed. (*: *p* < 0.05).

**iCAP treatment inhibits in vivo tumor growth in an NF1-associated MPNST xenograft mouse model.** Based on these results, we next evaluated the in vivo antitumor activity of iCAP. We utilized an NF1-associated MPNST cell line, S462TY (Figure 5a)**.** Longitudinal measurements of tumor volume demonstrated that tumor growth was abrogated in the treatment group, although this effect did not become statistically significant until Day 27 (Figure 5b and Figure S8). Tumor growth started to slow down in the CAP-treated group at day 10 post CAP exposure, but was not statistically significant. To confirm the mechanism involved in CAP treatment, the expression of the Ki67 protein and cleaved Caspase 3 was analyzed using immunohistochemical staining, which showed significantly increased cleaved Caspase 3 and attenuated Ki67 staining in the CAP-exposed xenografts (Figure 5c,d). 

## 4. Discussion

Although our current understanding of CAP and its anti-tumoral effects have yet to be fully elucidated, our studies indicate that NF1-associated PNST cell lines are sensitive to CAP-induced cell death. This observation has been made in other contexts, where increased CAP time exposure leads to greater cell death [20]. Previous work has shown that cells did not survive beyond 180 s of CAP exposure [21,22]. We have observed that apoptosis is a primary driver of cell death in these NF1 tumors following CAP exposure, similarly to other groups [23,24,25]. However, these cells may be remnants surviving after the initial CAP-triggered cell death, which was particularly evident after the 48 h threshold when assessing cell viability. Therefore, the observed reduction in cell proliferation could be attributed to a combination of induced cell death and cell cycle arrest, particularly in the MPNST cell line. This observation suggests the need for multiple consecutive CAP treatments to efficiently target cycling tumor cells in NF1-associated MPNSTs, but may not be necessary for NF1-associated PNs.

Interestingly, we noted that *NF1*-mutated cells were more sensitive to CAP-induced apoptosis than in the *NF1*-WT genetic background, and further work will need to assess the therapeutic window of CAP to avoid secondary toxicity, especially as our cell viability results demonstrated dose–response cellular death. Previous studies have targeted the generation of ROS in NF1-associated MPNSTs, but clinical trials have thus far not proven to be successful, likely due to therapeutic bioavailability [26,27]. The potential clinical applications of CAP are multifold, where it can be used as an adjunctive treatment to existing modalities and promote cancer cell death. Because NF1-associated PNSTs are heterogeneous, determining the appropriate dosage and frequency of CAP will likely need to be personalized.

For NF1-associated MPNSTs, our results demonstrate that Caspase 3, which is part of the extrinsic apoptotic pathway, is cleaved in the CAP-mediated in vivo setting [28]. To determine the mechanism of apoptotic cell death, we examined the cell cycle and found that CAP-mediated apoptotic cell death was more prominent in cells arrested in the G1 phase [29,30,31]. To that end, other groups have also demonstrated that CAP induces cell cycle arrest, pointing to CAP’s role in cell cycle mediation as a mechanism of cancer cell death [32,33,34]. Therefore, future studies could examine the synergistic role of current anti-cancer therapies and CAP in enhancing cancer cytotoxicity. 

Interestingly, we found that *NF1-*mutated cells were more sensitive to CAP-induced apoptosis in comparison to the wild type cells, including the human *NF1*-heterozygous Schwann cell line. *NF1* genetic mutations induce a hyperactive RAS/MEK/ERK pathway, which has led to the approval of selumetinib, a MEK inhibitor, for NF1-associated plexiform neurofibromas [35]. With the understanding that the MEK/ERK pathway can potentially be pro-apoptotic, there may be a lower threshold for apoptosis in *NF1*-mutated cells, although *NF1*-heterozygous cells do not have a hyperactive RAS/MEK/ERK pathway [36]. However, other groups have shown that CAP downregulates the MEK/ERK pathway to induce apoptosis [37,38]. Further studies are necessary to clarify the role of *NF1* mutations in CAP-induced apoptosis to refine the parameters of CAP treatment and its effect within different *NF1* gene mutation contexts. 

From an engineering standpoint, a translational hurdle to overcome is the ability to effectively monitor and regulate exposure that may affect the operation of CAP as well as determining the consistency and effectiveness of treatments in real time [39,40]. Several groups have attempted to introduce methods for feedback control and predictive modeling for CAP treatments to compensate for this variability [41]. As CAP is used in a large variety of applications, developing a control system that works across all fields has proven to be challenging. In addition, RONS are reactive, making these CAP-associated products difficult to detect in real time effectively and determine potential downstream mechanisms. In our studies, we utilized two experimentally established parameters for CAP treatment by using a CAP jet and helium as the carrier gas; however, further work is needed to develop standardized protocols that effectively translate this tool for widespread use in clinical settings [42,43].

Other limitations for CAP are the depth of delivery and dosage frequency for tumor treatment. Like the other groups’ findings, there was no significant difference in dCAP versus iCAP in the in vitro setting in NF1-associated tumors [44]. However, it is well known that dCAP can only induce apoptosis superficially due to penetration depth; our group has previously shown the use of a microneedle patch to address this constraint in the context of a melanoma [45,46]. In this study, we delivered PCM intratumorally and achieved anti-tumor effects in the in vivo setting. Although the difference in volume was not statistically significant until Day 27, one tumor in the control group was smaller, which may have affected the average volume in the control group. Nevertheless, given the efficacy of CAP in the in vitro setting, outstanding questions remain in uniformly delivering CAP in the context of individual clinical situations and determining PCM efficacy, as there is an inherent delay between CAP exposure and delivery [47]. Strategies to address these questions could include device development, such as laser interstitial thermal therapy (LITT) used for deep-seated intracranial lesions and CAP-containing nanoparticles.

## 5. Conclusions

To our knowledge, we are the first to demonstrate CAP as a potential treatment modality for NF1-associated PNSTs, and our findings are consistent with the findings of others that identify RONS-induced apoptosis as the mechanism by which CAP exerts its anti-cancer effects through the Caspase 3 pathway. This in vitro observation is supported by Ki67 attenuation in an established NF1-associated MPNST xenograft mouse model. One proposed mechanism by which cell death occurs upon CAP exposure is DNA damage leading to cell cycle arrest. Ultimately, this study offers evidence suggesting that CAP exposure could present a new therapeutic avenue for NF1-associated PNSTs, potentially complementing existing treatment modalities.

## Data Availability

The original contributions presented in the study are included in the article/Supplementary Materials; further inquiries can be directed to the corresponding author.

## Supplementary Materials

The following supporting information can be downloaded at: https://www.mdpi.com/article/10.3390/biomedicines12091986/s1. Figure S1. Positive control with staurosporine, a known inducer of apoptosis. All experiments were done in quadruplicate. (A) The ESC-FC1208NF1 (Nf1^−/−)^ and ESC-FH1208NF1 (Nf1 wild type) mouse Schwann cell lines. (B) NF1^+/−^ Schwann cell and NF1 wild type Schwann cell. (C) Patient derived NF1-mutated plexiform neurofibromas (D) NF1-associated MPNSTs (S462TY and 88-14) and a sporadic MPNST (STS26T). Figure S2. Negative control with non-CAP exposed cells. All experiments were done in quadruplicate. (A) The ESC-FC1208NF1 (Nf1^−/−^) and ESC-FH1208NF1 (Nf1 wild type) mouse Schwann cell lines. (B) NF1^+/−^ Schwann cell and NF1 wild type Schwann cell. (C) Patient derived NF1-mutated plexiform neurofibromas (D) NF1-associated MPNSTs (S462TY and 88-14) and a sporadic MPNST (STS26T). Figure S3. (a) S462TY cells in G1 phase, (b) S462TY cells in S phase, (c) S462TY cells in G2/M phase, (d) S462TY cell debris showing dead cells. Figure S4. Flow cytometry analysis for S462TY cell line. (A) Control at 12 h mark with standard medium. (B) 12 h after PCM transfer. (C) Control at 24 h mark with standard medium. (D) 24 h after PCM transfer. Figure S5. Flow cytometry analysis for CRL-3392 (wild type Schwann cell). (A) Control at 12 h mark with standard medium. (B) 12 h after PCM transfer. (C) Control at 24 h mark with standard medium. (D) 24 h after PCM transfer. Figure S6. Flow cytometry analysis for CRL-3391 (NF1-heterozygous Schwann cell). (A) Control at 12 h mark with standard medium. (B) 12 h after PCM transfer. (C) Control at 24 h mark with standard medium. (D) 24 h after PCM transfer. Figure S7. Flow cytometry analysis for CRL-3387 (NF1-mutated plexiform neurofibroma). (A) Control at 12 h mark with standard medium. (B) 12 h after PCM transfer. (C) Control at 24 h mark with standard medium. (D) 24 h after PCM transfer. Figure S8. Average of actual volume of treated versus control tumors for the in vivo experiment at Day 0 prior to iCAP or PBS exposure, respectively.
