# Supplementary material for: Cold Atmospheric Plasma Induces Growth Arrest and Apoptosis in Neurofibromatosis Type 1-Associated Peripheral Nerve Sheath Tumor Cells
Source: Biomedicines. 2024 Sep 2;12(9):1986. doi: 10.3390/biomedicines12091986 (PMC11429496; doi:10.3390/biomedicines12091986)

## Slide 1
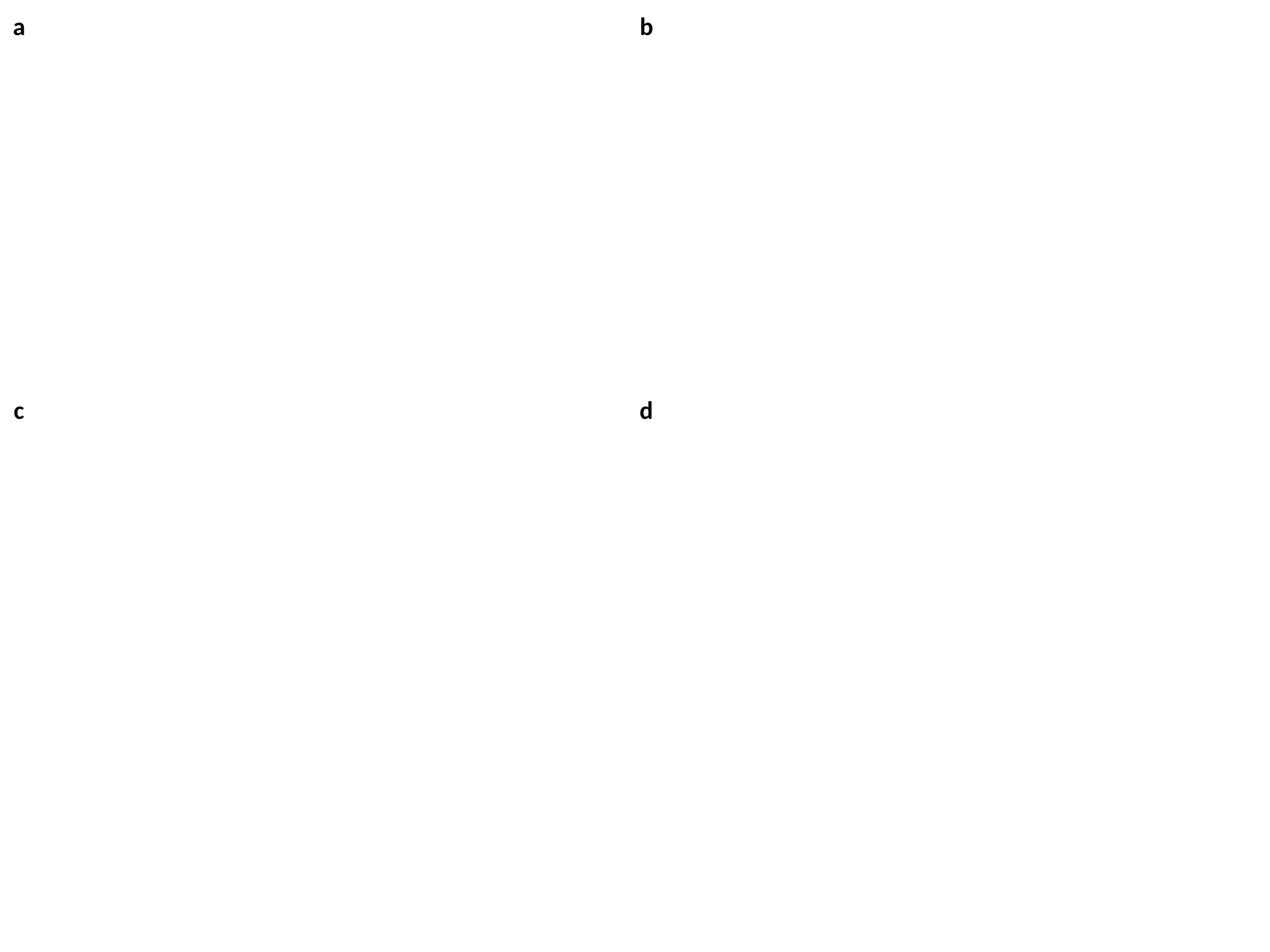

a
b
c
d

## Slide 2
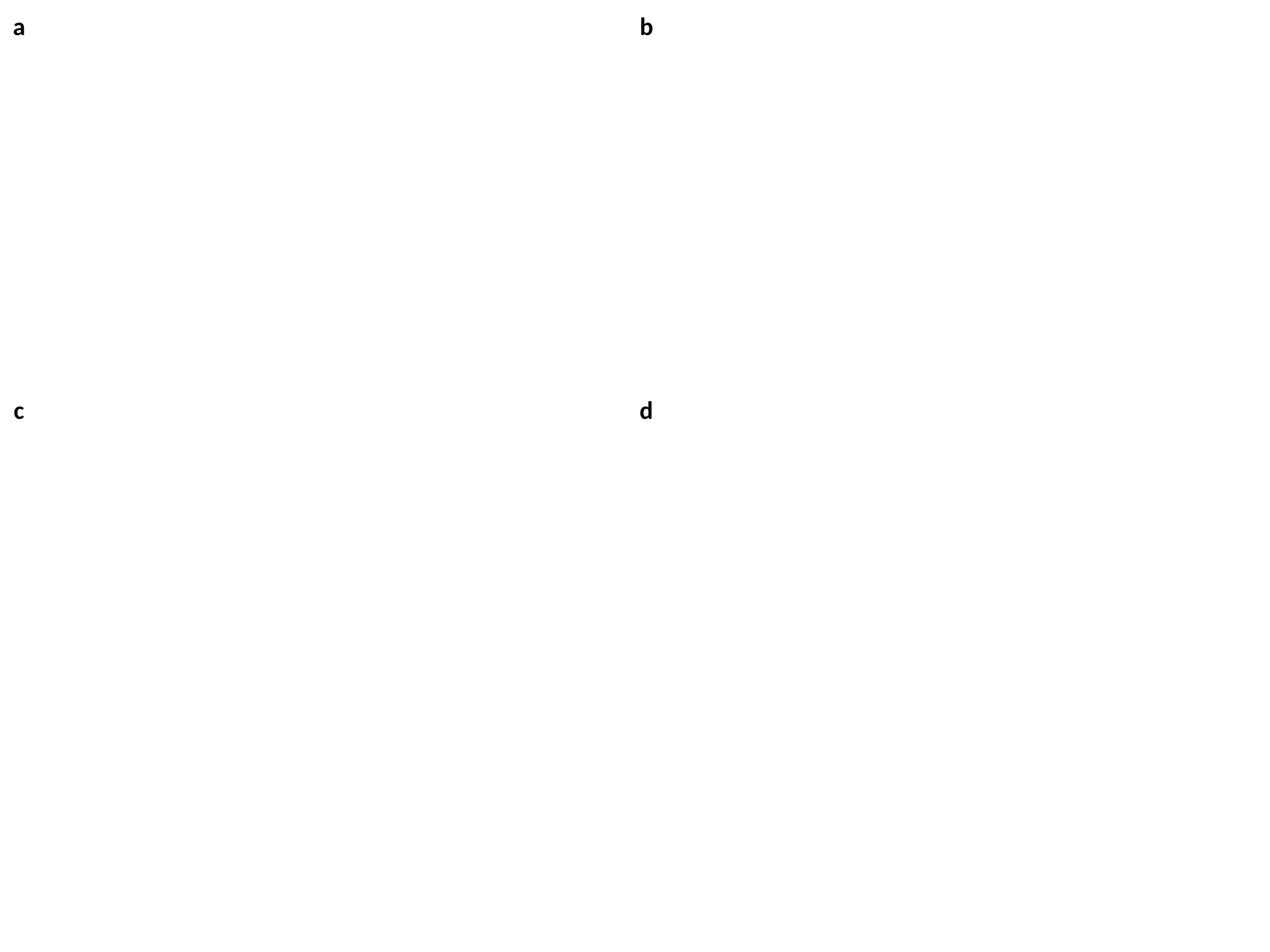

a
b
c
d

## Slide 3
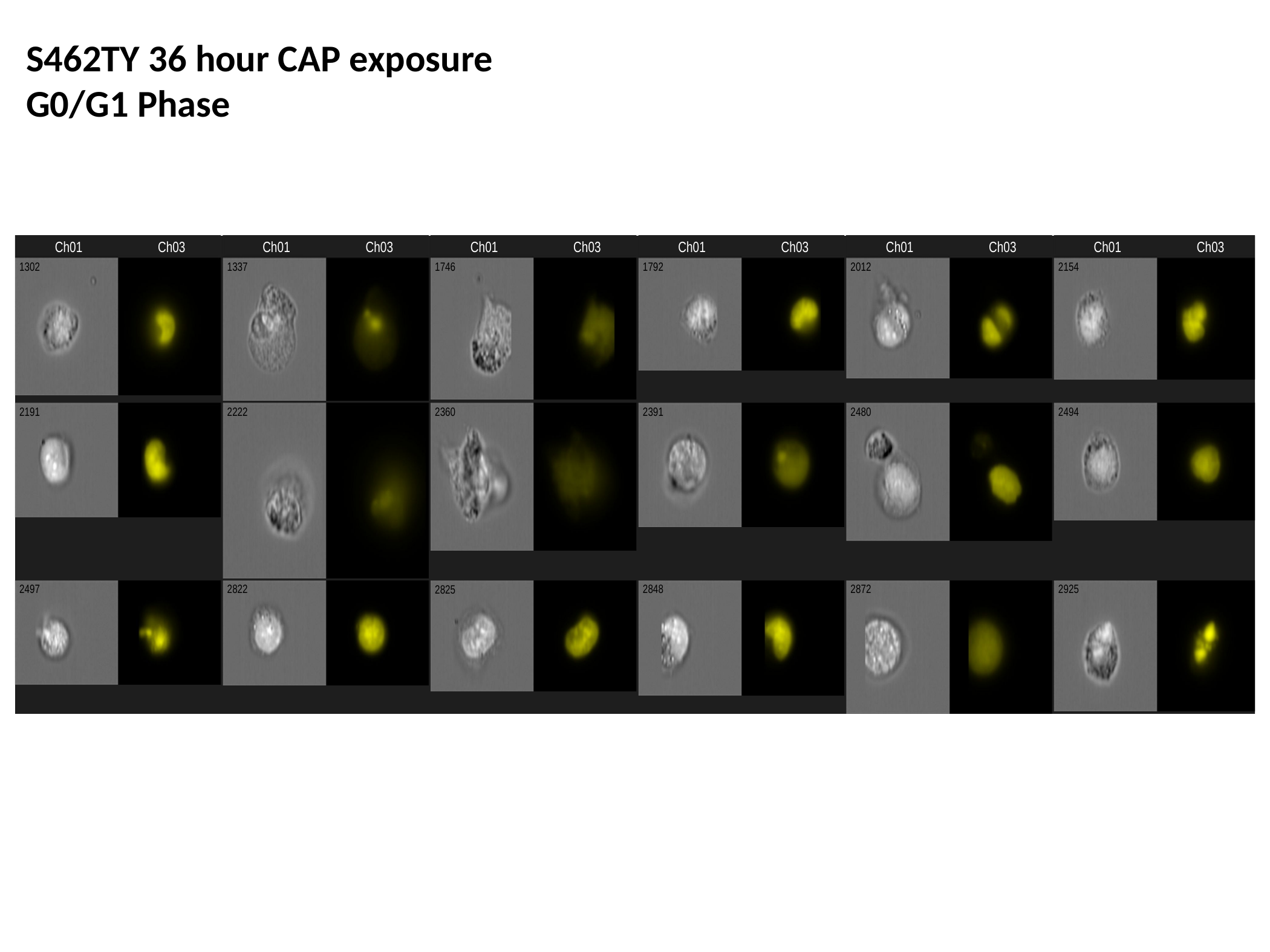

S462TY 36 hour CAP exposure
G0/G1 Phase

## Slide 4
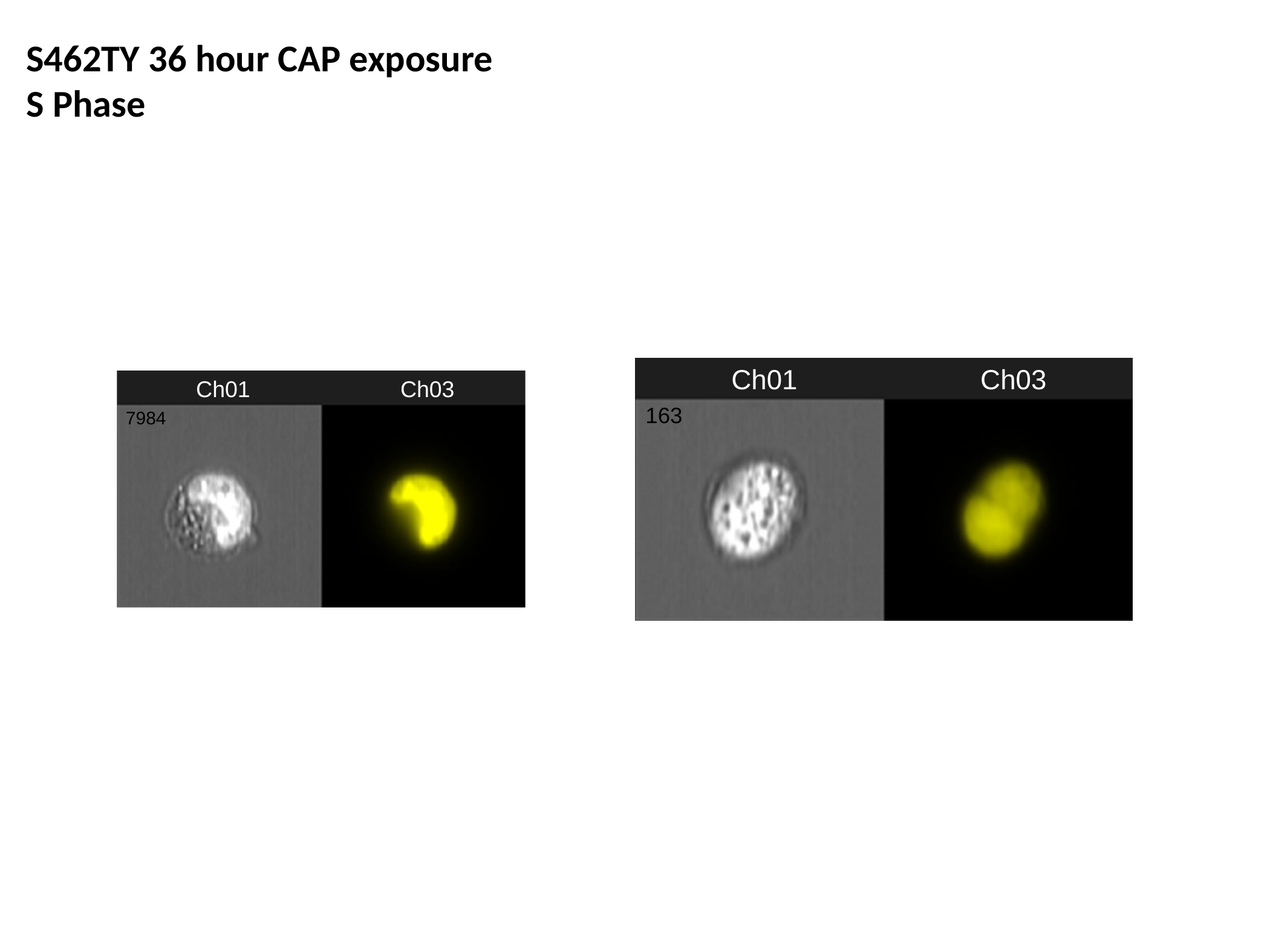

S462TY 36 hour CAP exposure
S Phase

## Slide 5
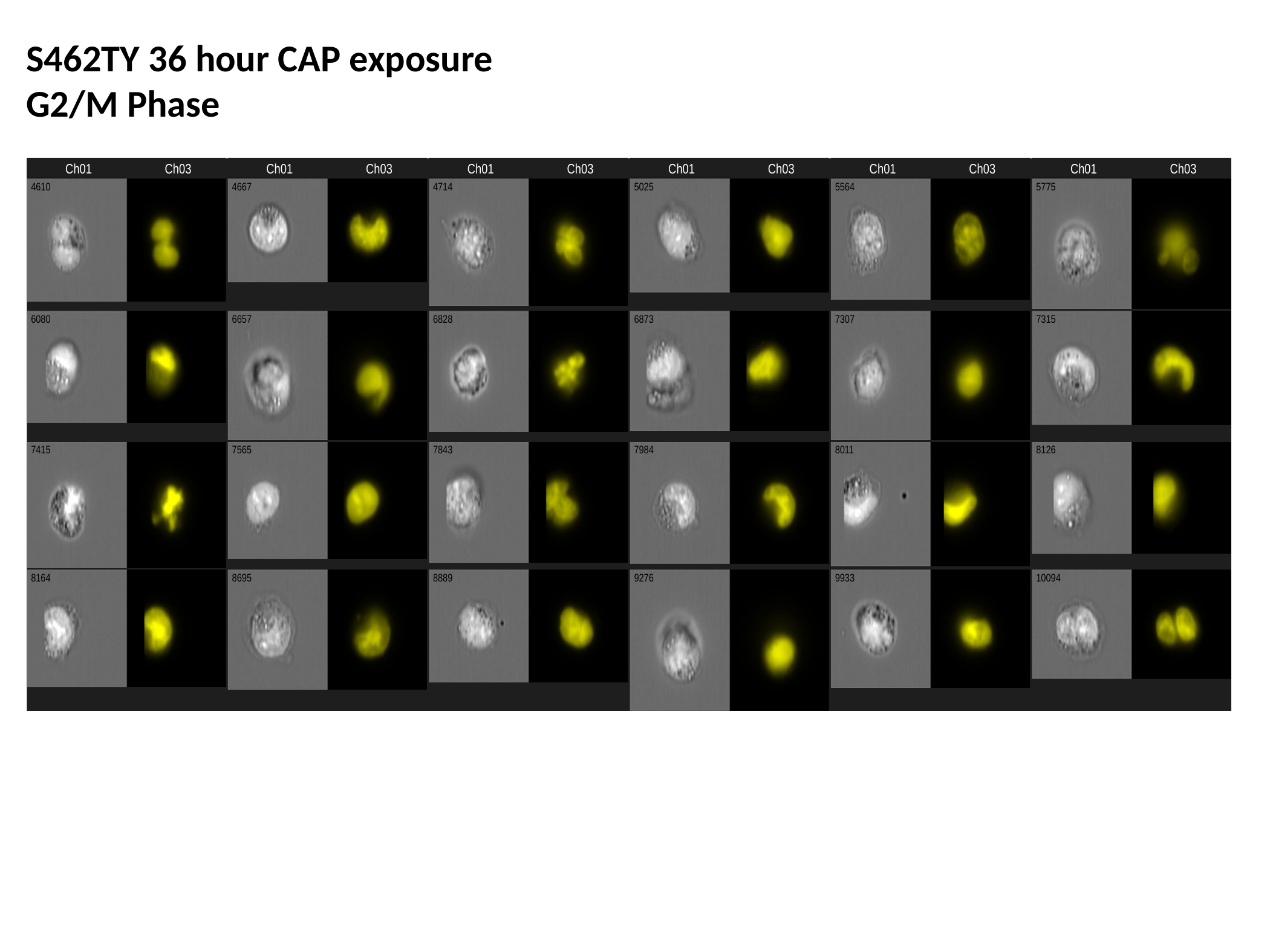

S462TY 36 hour CAP exposure
G2/M Phase

## Slide 6
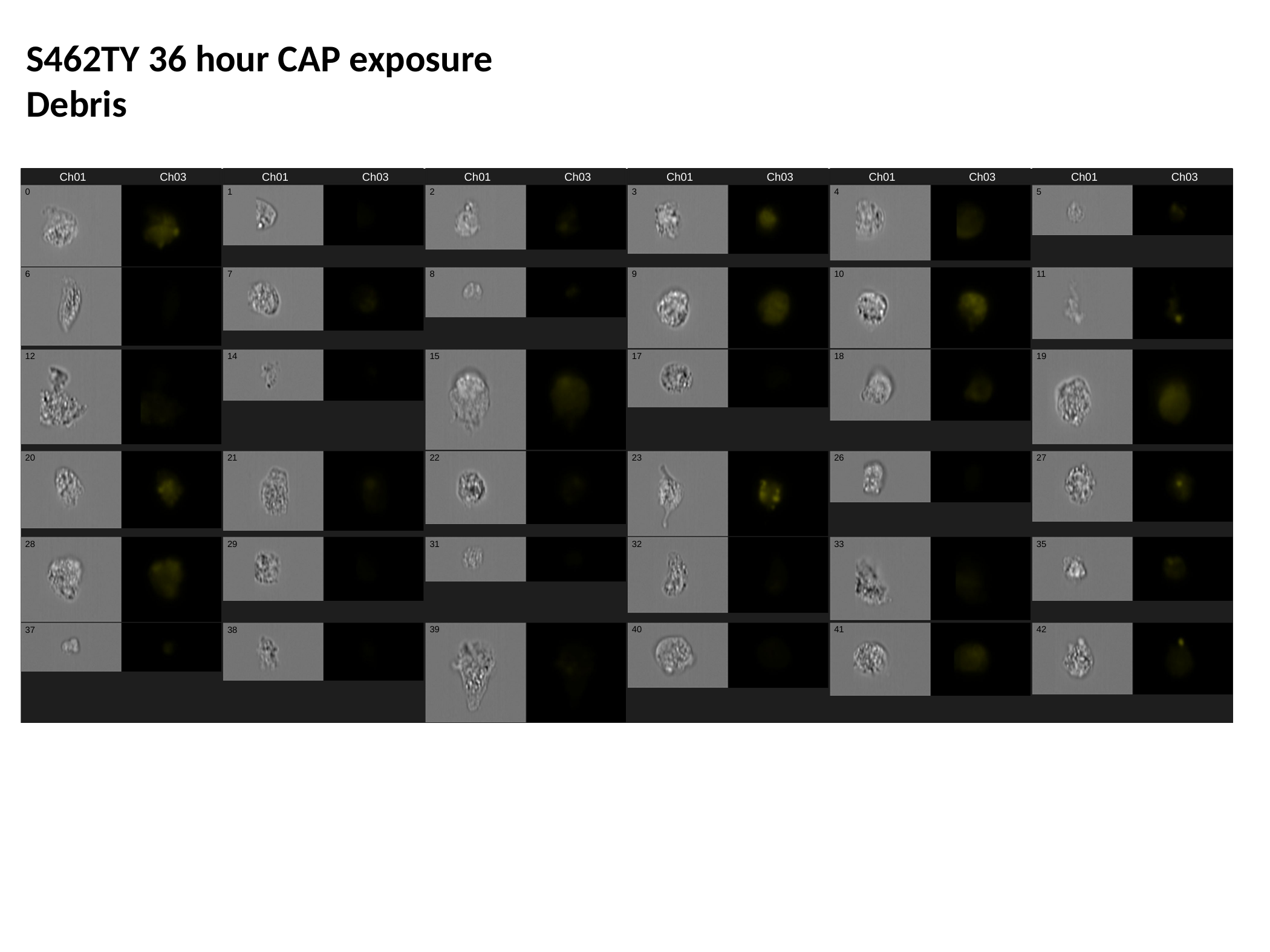

S462TY 36 hour CAP exposure
Debris

## Slide 7
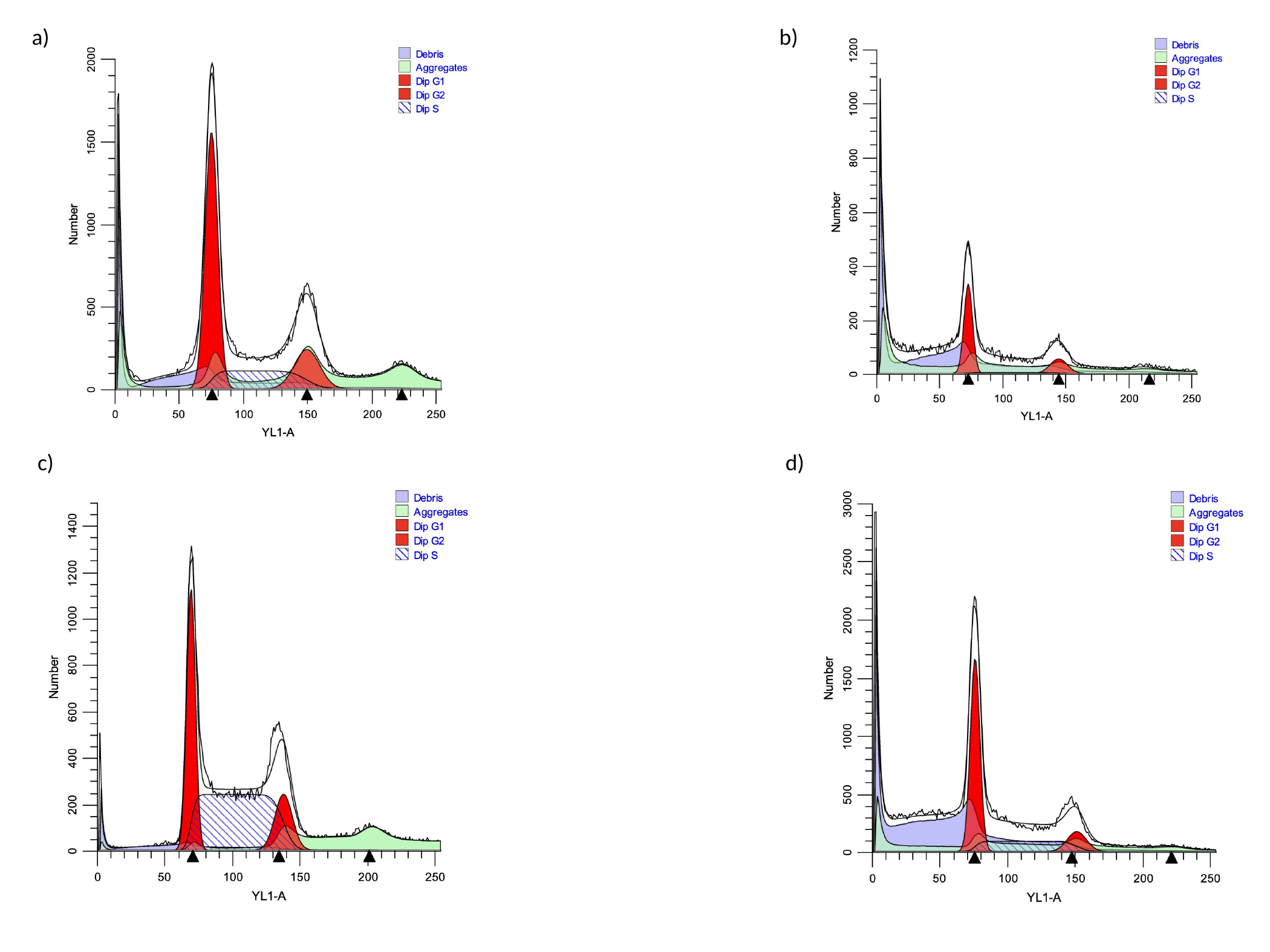

a)
b)
c)
d)

## Slide 8
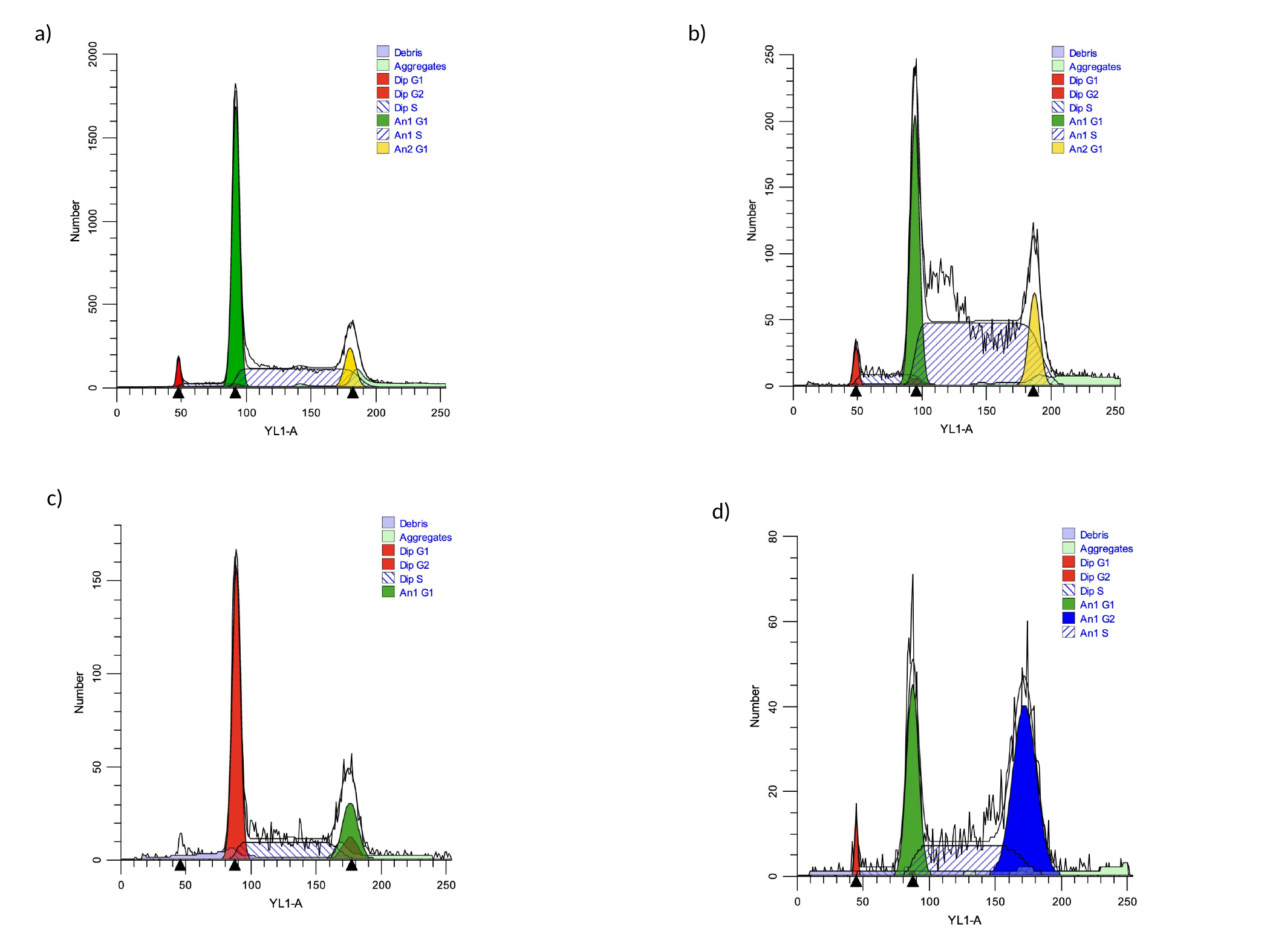

a)
b)
c)
d)

## Slide 9
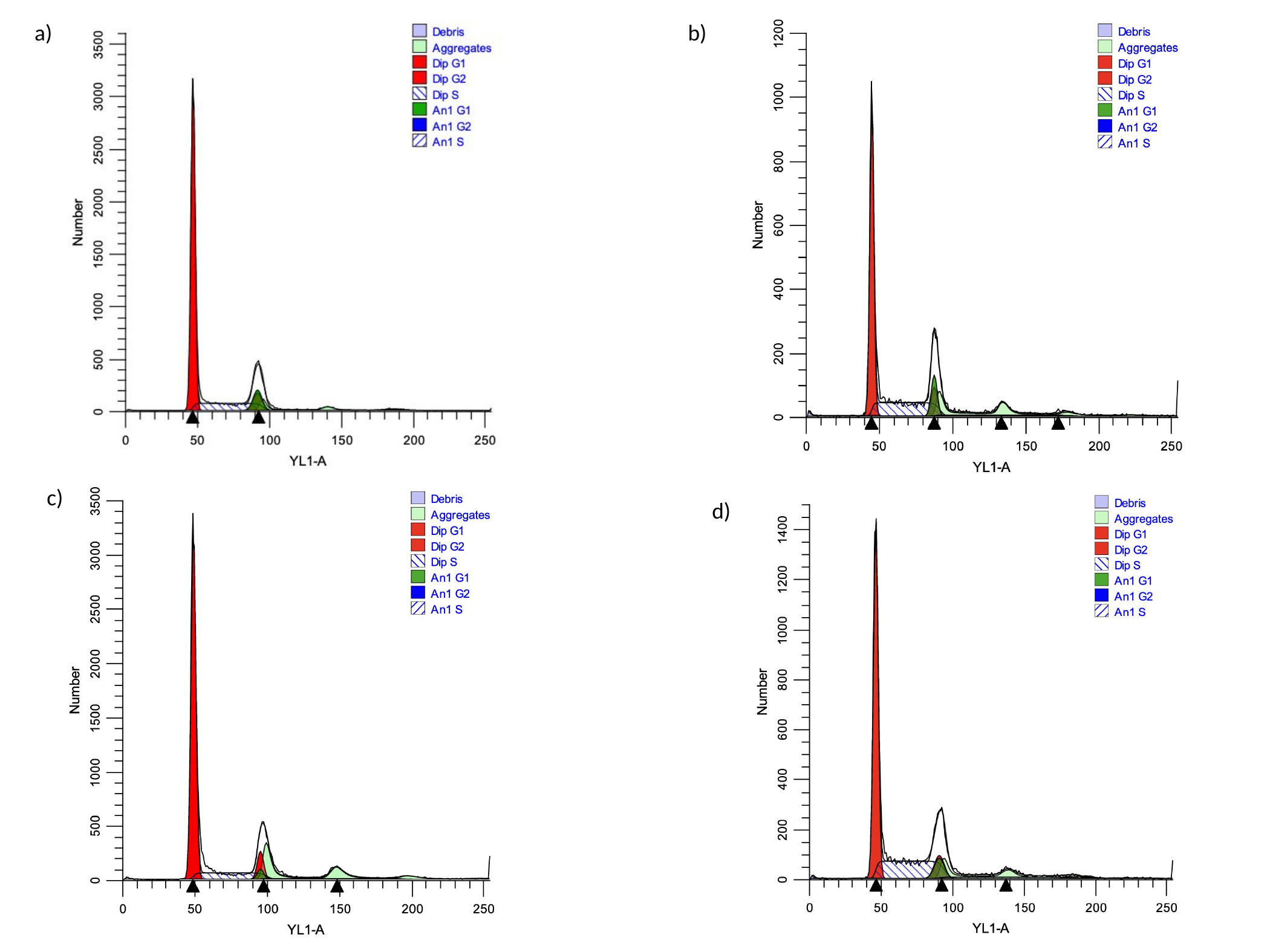

a)
b)
c)
d)

## Slide 10
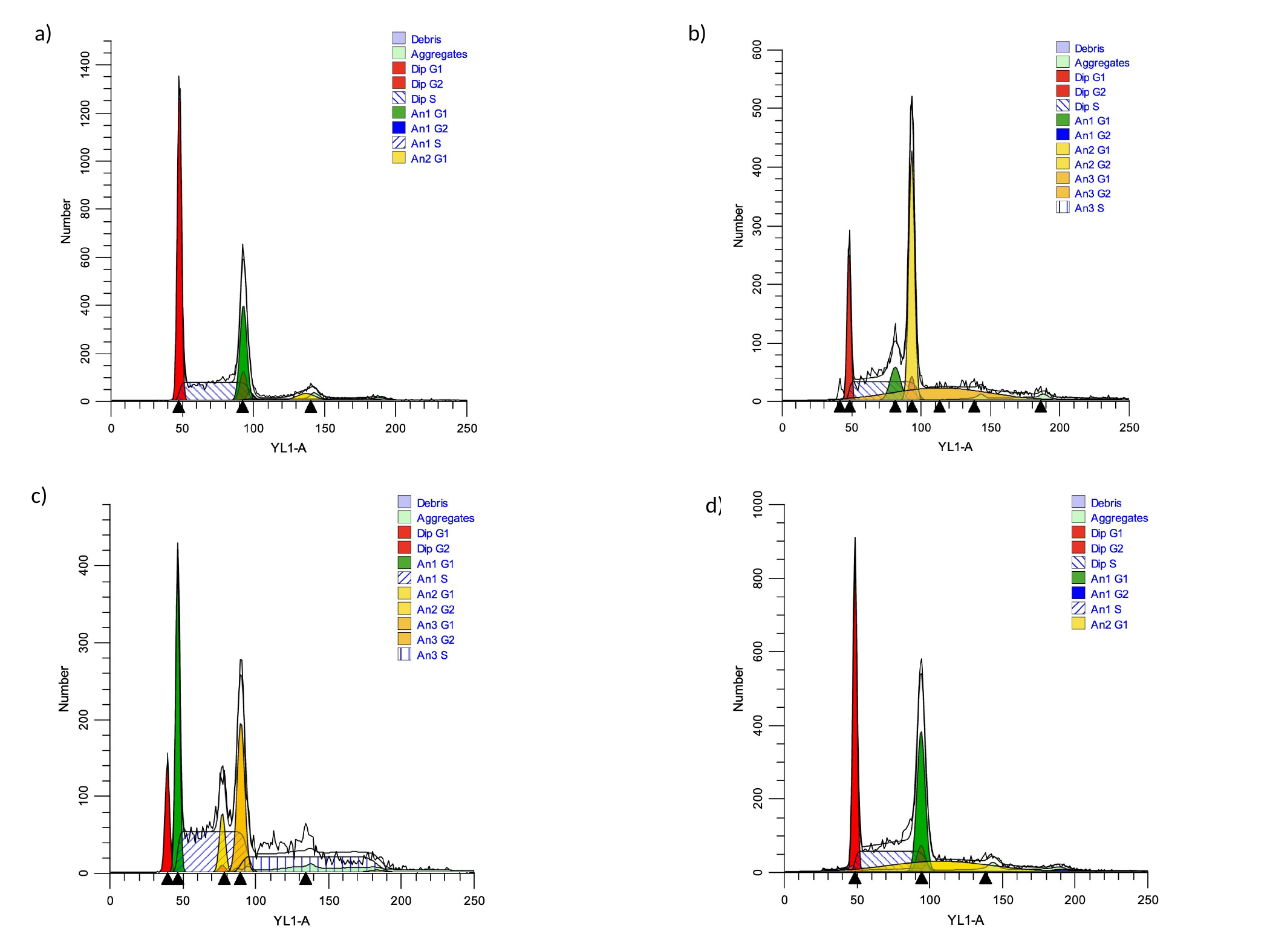

a)
b)
c)
d)

## Slide 11
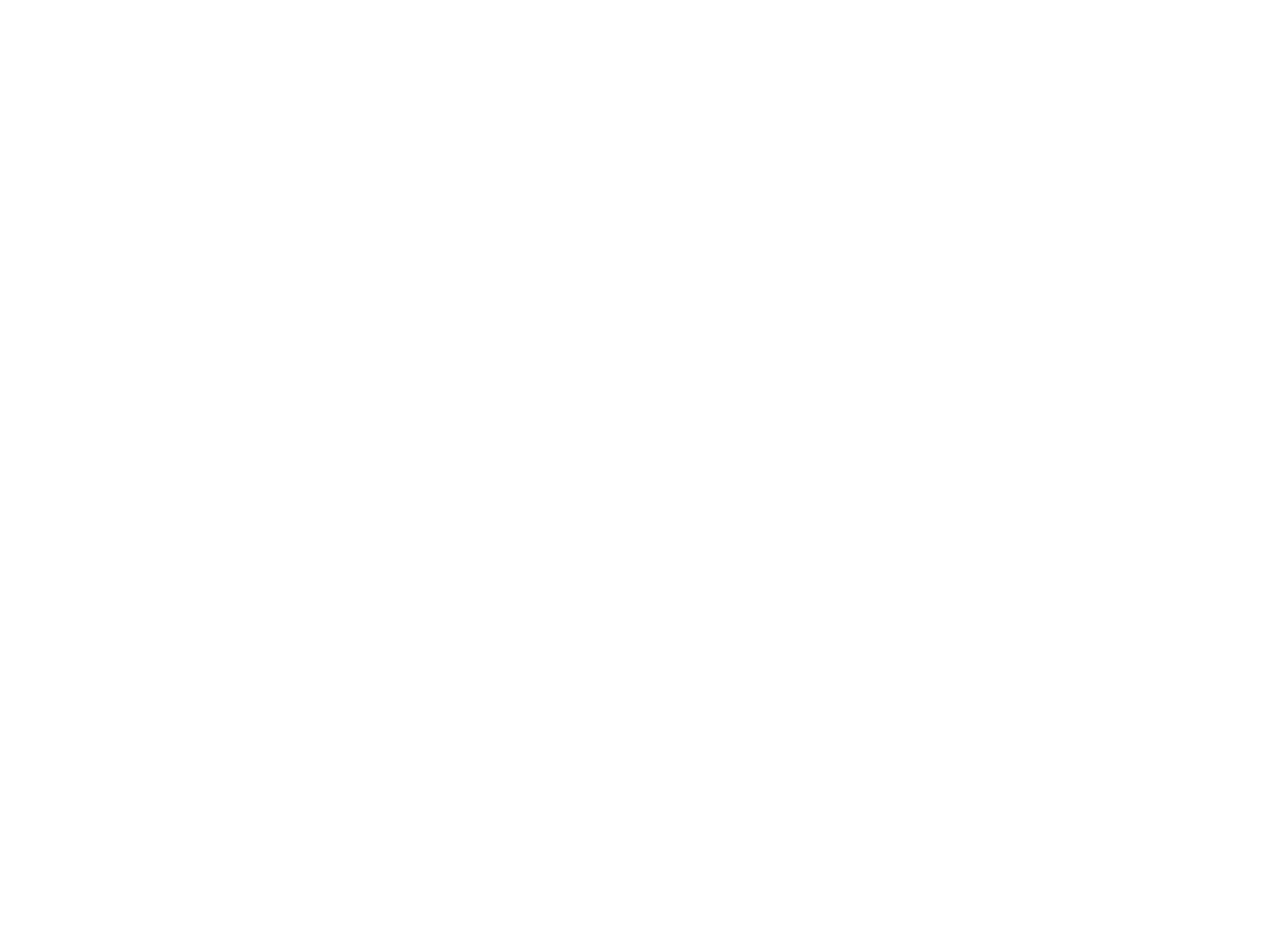

Supplement: Supplementary file 1 [file biomedicines-12-01986-s001.zip › 20240514 Supplementary Figures.pptx]
